# Supplementary material for: Number of antenatal care utilization and associated factors among pregnant women in rural Ethiopia: Zero-inflated Poisson regression of 2019 intermediate Ethiopian Demography Health Survey
Source: PLoS One. 2024 Nov 25;19(11):e0311299. doi: 10.1371/journal.pone.0311299 (PMC11588278; doi:10.1371/journal.pone.0311299)
Supplement: Attachment — Submitted filename: Peer_Review final 2.docx [file pone.0311299.s001.docx]

**Date:** Wednesday, June 12th, 2024

**Journal:** PLOS ONE

Dear Editor-in-Chief, Associate Editor, and Authors

Thank you for the opportunity to review the manuscript entitled "Number of antenatal care utilization and associated factors among pregnant women in rural Ethiopia: Zero-inflated Poisson regression of 2019 intermediate Ethiopian Demography Health Survey ".

- **Summary:**

In this study, the authors conducted a comprehensive analysis using Zero-inflated Poisson regression to explore the antenatal care utilization in Ethiopia, alongside the associated factors influencing the accessibility of Ethiopian pregnant women. This research sets itself apart from existing literature by focusing specifically on rural areas of Ethiopia, and considering the demographic characteristics of the women themselves. Notably, factors such as socioeconomic status, geographical distribution and constraints, and parity were scrutinized to provide a comprehensive understanding of ANC utilization dynamics. Nevertheless, healthcare infrastructure was not highlighted well.

To gather data, the authors utilized information from the 2016 Ethiopian Demographic and Health Survey, ensuring a robust sample size for their analysis to draw a reliable conclusion.

The significance of this study lies not only in its transparent analysis of data but also in its potential to inform targeted interventions and policy reforms. By shedding light on the specific challenges faced by pregnant women in rural Ethiopia and identifying key determinants of ANC utilization, this research serves as a vital milestone for healthcare policymakers, offering actionable insights to catalyze positive change and enhance the delivery of maternal healthcare services nationwide.

In summary, the authors have identified a wide range of socioeconomic, demographic, and geographical factors that play a significant role in determining the utilization of antenatal care. These findings have been effectively presented through informative tables, enhancing the clarity and understanding of the study's results.

- **Comments to Authors:**

**Abstract:**

- please correct the number stated in line 42, written in bold in the following sentence (The highest percentage (29.65%) of antenatal care visits was observed from 25 to **2age.**)

**Title:**

- The title of the study is clear, inclusive and precise to the study’s objectives and aims. The authors stated the study design distinctly.

**Introduction:**

- The introduction is well structured, clear, and straightforward. The authors had effectively introduced the concept of antenatal care in alignment with the WHO FANC strategy, and highlighted the importance of regular ANC in promoting the health of mothers and their newborns (live birth weight in specific).
- The rationale and aim of the study are well stated.
- The relevant statics sourced from the WHO and the 2016 Ethiopian Demographic and Health Survey by the authors have established a robust citation that contributes significant value.

**Methods:**

- The authors conducted a database study.
- The authors effectively described the geographical location and administrative divisions of Ethiopia, which provides more comprehension for the non-Ethiopian audience.
- The section provides a detailed overview of the study design, setting, data collection methods, and sample size determination. In addition, they included essential information, needed for further understanding of the analytical framework. However, mentioning some potential challenges in choosing method of analysis would be a qualitative addition.
- Concerning the data analysis, the authors have successfully mentioned the source of data and the justification for accessing it, which adds transparency to the research process.

**Results:**

- The authors conducted a thorough analysis of the results, supported by sufficient amount of data justifying the conclusion. Nevertheless, the analysis assumed a linear relationship between variables, overlooking potential confounding factors that could impact the accuracy of correlations. Therefore, further research may be warranted to validate these hypotheses.
- The authors sub-categorized the study variables, which provided a more inclusive perspective.

**Discussion:**

- In the discussion section, the authors have effectively compared their findings with those of similar studies and offered reasonable explanations for both the similarities and differences.
- Furthermore, the authors did highlight the study's limitations in a transparent discussion of these limitations.
- Further clinical application might be mentioned for policy makers, together with future direction and recommendation.

**Conclusion:**

- Correctly answered the research question.
- The authors were able to mention the most important research results in a concise and clear manner.
