# Supplementary material for: Number of antenatal care utilization and associated factors among pregnant women in rural Ethiopia: Zero-inflated Poisson regression of 2019 intermediate Ethiopian Demography Health Survey
Source: PLoS One. 2024 Nov 25;19(11):e0311299. doi: 10.1371/journal.pone.0311299 (PMC11588278; doi:10.1371/journal.pone.0311299)
Supplement: Attachment — Submitted filename: Response to Reviewers.docx [file pone.0311299.s002.docx]

**Response to reviewers point by point**

First of all, I would like to acknowledge your collective concern, time, devotion, patience, and valuable comments. Your insights have greatly contributed to improving my understanding of the area.

**Reviewer comment #1:**

It’s a very well written and structured article. All the sections including abstract, methodology, results and discussion are very well described. The results are displayed in detail and elaborate.

**Author response:** Thank you for your kind words and detailed feedback. I am pleased to hear that you found the article well-written and structured, with clear descriptions and detailed results. Your comments are greatly appreciated.

**Reviewer comment #2:**

1. Please correct the number stated in line 42, written in bold in the following sentence (The highest percentage (29.65%) of antenatal care visits was observed from 25 to 2age.)

**Author response:** Thank you for pointing out the discrepancy. I have reviewed the result section and corrected the percentage. The revised statement now accurately reflects that the highest percentage (29.65%) of antenatal care visits was observed among women aged 25-29 years.

2. The section provides a detailed overview of the study design, setting, data collection methods, and ample size determination. In addition, they included essential information, needed for further understanding of the analytical framework. However, mentioning some potential challenges in choosing method of analysis would be a qualitative addition.

**Author response:** Thank you for your feedback. I have added a discussion of potential challenges associated with the chosen method of analysis to provide a more comprehensive overview. This addition addresses possible issues related to the analytical approach and enhances the understanding of its application in the study.

3. The authors conducted a thorough analysis of the results, supported by sufficient amount of data justifying the conclusion. Nevertheless, the analysis assumed a linear relationship between variables, overlooking potential confounding factors that could impact the accuracy of correlations. Therefore, further research may be warranted to validate these hypotheses.

**Author response:** Thank you for your insightful comment. You are correct that the analysis assumed linear relationships between variables, and potential confounding factors may impact the accuracy of the correlations. The 2019 Ethiopian Mini DHS data used in our study did not include some variables that could influence the outcome, which may have limited our analysis. We acknowledge this as a limitation of using the Mini DHS data. Further research incorporating a broader set of variables and exploring potential confounders would be valuable to validate and refine these hypotheses.

4. Further clinical application might be mentioned for policy makers, together with future direction and recommendation.

**Author response:** Thank you for your suggestion. I have thoroughly revised the manuscript to include further clinical applications for policymakers. Additionally, I have added on the last part of my discussion on future directions and recommendations to provide more detailed guidance for subsequent research and practice.

**Reviewer comment #3:**

The study is well formatted and quite informative on the level of health care throughout the country, good analysis of the data of ANC depending on different variables including geography, religion, education and socio-economy, make the study is more reflecting the culture in concern.

**Author response:** Thank you for your thoughtful comments and positive feedback. I appreciate your recognition. Your insights are valuable and encouraging.

**Reviewer comment #4:**

1. Table on competing interest typographical error written as "no computing of interest"

**Author response:** First of all thank you for your time and comments. The “Competing interests” is written as “The authors declare that they have no competing interests.”

2. Ethics statement should be more robust. the NHS data that was used should be well spelt out

**Author response:** Thank you for your feedback. I have revised the ethics statement to provide a more comprehensive account. The study utilized secondary data from the National Health Survey (NHS), which is publicly available and does not require individual ethics approval for use. We accessed the data through the DHS (Demographic and Health Surveys) web archive by registering and obtaining permission to download the datasets. All relevant ethical guidelines and regulations for secondary data use were strictly followed to ensure the integrity of the research.

3. The result in the abstract and that in the method result and findings are different. Likewise, the number of women studied were different 2970 and 2896.7 were quoted. Since no of pregnant women are whole numbers, the value of 2896.7 is not appropriate.

**Author response:** Thank you for pointing out the discrepancies. The number of women included in the study, as shown in Figure 1, is indeed 2,970. The figure 2,896.7 refers to the weighted number of women, obtained using the DHS (Demographic Health Surveys) data. To ensure representativeness, we applied a weighting factor using the Stata command generate wt=v005/1000000. The resulting weighted value, 2,896.7, represents the statistical adjustment rather than an actual fraction of individuals. We have clarified this distinction by separately presenting the "weighted" and "unweighted" figures in the results section to avoid confusion.

4. Were the women interviewed to get the number of ANC visits they had or all data were taken from the NHS data. This is important as line 371-372 stated the factor of recall bias. If the data has been recorded, there should not be recall bias.

**Author response:** Thank you for raising this important point. The data for the study was sourced entirely from the DHS data, and the number of antenatal care (ANC) visits was not collected through direct interviews. According to the "***Guide to DHS Statistics, DHS-7 (version 2)***," the data includes:

**Numerators**:

The number of women who received antenatal care for their last birth, categorized by the number of visits (***variable m14***).

The number of women who received antenatal care for their last birth, categorized by the number of months pregnant at the time of the first visit (***variable m13***).

**Denominator**: The number of women who had a birth in the last five years (***variable midx = 1***).

In the analysis, we focused on women whose last birth occurred within the past five years (midx = 1), which helps in ensuring the data's relevance. Despite this, recall bias can still occur, particularly for women who may find it challenging to remember the exact number of ANC visits or the timing of their first visit, especially if their last pregnancy was several years ago. This potential recall bias was noted in the discussion to account for variations in memory accuracy over time.
